# Effect of wheat germ on metabolic markers: a systematic review and meta-analysis of randomized controlled trials

**DOI:** 10.1007/s10068-020-00769-9

**Published:** 2020-07-14

**Authors:** Humna Liaqat, Eunseon Jeong, Kyeong Jin Kim, Ji Yeon Kim

**Affiliations:** 1 Department of Food Science and Technology, Seoul National University of Science and Technology, 232, Gongneung-ro, Nowon-gu, Seoul, 01811 Korea; 2 Department of Nano Bio Engineering, Seoul National University of Science and Technology, 232, Gongneung-ro, Nowon-gu, Seoul, 01811 Korea

**Keywords:** Wheat germ, Metabolic markers, Cholesterol, Triglycerides, Glucose

## Abstract

This systematic review and meta-analysis aim to evaluate the association of wheat germ interventions and metabolic markers. An electronic search was performed by mid-May 2019 in the PubMed, Google Scholar, and Web of Science databases. Quality was evaluated using the risk of bias assessment tools. Thirty-three randomized controlled trials (RCTs) were identified, among which ten were suitable and systematically reviewed based on biomarkers (cholesterol, triglycerides, glucose, and oxidative stress). Three biomarkers in five eligible studies were investigated by meta-analysis. Total cholesterol showed non-significant results (*p* = 0.98), with standard mean difference (SMD) of − 0.01 (95% confidence interval; − 0.17, 0.16). The SMD was − 0.06 (95% CI − 0.41, 0.29, n = 4) for triglycerides and − 0.09 (95% CI − 0.62, 0.45, n = 2) for glucose. No biomarkers showed heterogeneity (0%). This review revealed non-significant association between wheat germ interventions and metabolic markers. Sensitive analysis with high-quality RCTs may be worth trying.

## Introduction

Metabolic syndrome (MetS) is an asymptomatic disorder that includes a cluster of metabolic abnormalities associated with obesity, hyperlipidemia, hypertension, and insulin resistance (Alberti et al., 2009). The causative factors of MetS are central obesity and insulin resistance, which lead to cardiovascular diseases (CVDs), diabetes, and stroke (Srikanthan et al., 2016). Oxidative stress and inflammation also contribute to the etiology of MetS (Soares and Costa, 2009). Metabolic markers such as triglyceride levels, high-density lipoprotein-cholesterol (HDL-C), low-density lipoprotein-cholesterol (LDL-C), hypertension, blood pressure, obesity, insulin, and oxidative stress are the criteria used to diagnose MetS. This non-communicable disease has become a significant major cause of mortality worldwide and increases the mortality rate of patients with type 2 diabetes and CVDs, coronary heart disease, and stroke (Ford, 2004). The American Heart Association reported that about 35% of adults and 50% of 60 years older in the US have MetS (Aguilar et al., 2015). The International Diabetes Federation stated that nearly 25% of the world’s population suffers from MetS (O’neill and O’driscoll, 2015). However, the prevalence varies by age, ethnicity, gender, and variation in the definition of MetS. Based on the International Diabetes Federation definition, the eastern country of Tunisia showed a MetS prevalence of 45.5%; in Iran, this value was 37.4% (Delavari et al., 2009).

Recently, many clinical studies have been conducted to evaluate the relationship between unhealthy dietary habits and chronic diseases such as CVD (Michas et al., 2014; Willett et al., 2002) and diabetes (Esposito et al., 2015; Hauner et al., 2012). The inclusion of functional components in diets plays an integral role in the public health sectors (De Jong et al., 2004; Vella et al., 2014). Refined grains are extracted from cereals by removing the bran and germ fractions. These fractions contained bioactive compounds such as phytochemicals, some essential micronutrients, vitamins, and dietary fiber. Many studies have demonstrated an association between CVD and whole grain and bran consumption (Aune et al., 2016; Charlton et al., 2012; Junejo et al., 2019; Zong et al., 2016). However, the results for germ are unclear (de Munter et al., 2007; Lupton et al., 1994).

Wheat (*Triticum aestivum*) is one of the most widely consumed edible whole grains worldwide and is used as a staple food in many countries. Wheat is comprised of nearly 80% endosperm, 15% bran, and 5% germ (Slavin, 2004). Wheat germ (the embryo) is a concentrated source of antioxidants such as polyphenols, carotenoids, and tocopherols (the most abundant natural source of vitamin E) (Vaher et al., 2010; Zhu et al., 2011). Wheat germ proteins are ample sources of amino acids, especially methionine, threonine, and lysine (Meriles et al., 2019). Wheat germ is typically discarded during the milling process but has been used to produce wheat germ oil. In the previous decade, numerous in vitro and in vivo studies have investigated the various health aspects of wheat germ, especially wheat germ oil (Arshad et al., 2013; Khedr, 2017) that can improve lipid metabolism (Khalil et al., 2010) and lower oxidative stress (Alessandri et al., 2006). Fermented wheat germ extract (FWGE) has been shown to have antimetastatic effects in cells and animals (Fajka-Boja et al., 2002; Heimbach et al., 2007; Hidvegi et al., 1998) including in colorectal (Farkas, 2005) and ovarian cancer (Koh et al., 2018). Many in vivo trials have been conducted to determine the preventive role of wheat germ on atherosclerosis, hypercholesterolemia (Rezq and Mahmoud, 2011), hyperlipidemia (Chadha et al., 2015), oxidative stress (El-Shorbagy, 2017), hepatotoxin (Akool, 2015) and insulin resistance (Iyer and Brown, 2011; Ojo et al., 2017). Some in vitro studies demonstrated the antioxidant and anti-inflammatory effects of wheat germ and wheat germ oil (Boros et al., 2001; Jeong et al., 2017; Park et al., 2015).

Hence, after reviewing numerous studies, this comprehensive systematic review aims to summarize the accessible scientific literature on wheat germ regarding its effectiveness with metabolic markers in humans.

## Materials and methods

We carried out this systematic review and meta-analysis in accordance with the PRISMA statement (Moher et al., 2009) and Cochrane Collaboration (Higgins and Green, 2011) during all stages of execution and data reporting.

### Literature search

A comprehensive search strategy was applied by using the medical and electronic databases Google Scholar, Medline (PubMed), and Web of Science without any restrictions on language or time to identify articles published by mid-May 2019. Research articles using “wheat germ” in the title and abstract were searched. To obtain more precise results, an advanced search was conducted with filters such as clinical trials, species (human) examined, and terms including “wheat germ” OR “randomized” OR “controlled trials”. To evaluate whether wheat germ is related to MetS, we identified the studies of wheat germ and metabolic markers using the terms cholesterol, glucose, oxidation, triglycerides, lipids, obesity, and blood pressure in combination with wheat germ. We screened additional review and systematic review studies to identify potentially related citations. Manual searching was performed to avoid the elimination of pertinent articles.

### Study selection and eligibility criteria

This review was limited to randomized controlled trials (RCTs, either parallel or crossover) conducted solely in adult humans. PICOS (population, intervention, comparator, outcome, and study design) was established for the review. Eligibility criteria were based on the PICOS reporting tools (Methley et al., 2014). The study population included healthy persons or people who were at risk of disease occurrence such as pre-diabetes and impaired fasting glucose. Study interventions included wheat germ in the raw, extracted, powder, or oil forms that evaluated the effect of wheat germ in reducing the MetS by lowering its biomarkers like blood glucose, cholesterol, lipid contents, blood pressure, and overweight (obesity). The intervention was compared to control or placebo groups in a single or double-blinded manner. If any studies fulfilled these eligibility criteria, they were included in the systematic review regardless of the availability of analytical data for meta-analysis. The following studies were excluded from analysis: those in which participants had a disease, RCTs that did not report the effect of wheat germ on any metabolic markers, in vivo (non-human studies) and in vitro studies, papers with the abstract only, conference abstract, and observational, coherent, and case–control studies. In the selection process, all controversies and disagreements were resolved by discussion among the two investigators.

### Data extraction

In the initial search, two researchers (HL and EJ) independently reviewed the title and abstracts of the articles under the PICOS framework. Next, descriptive data screened based on full-text articles were assessed for eligibility. A standard form included the following information from the selected articles: bibliographic details, study design, study origin, participants’ health status, age, sex, body mass index, groups description, a form of wheat germ, intervention period, washout period, dose amount, intake direction, physical and dietary intake details during an intervention, functionality of wheat germ, biomarker readings at baseline and post-intervention, outcomes measures, statistical results, compliance, and dropout rate.

There were insufficient data on dichotomous outcomes in the included studies. To utilize the available data in a meta-analysis, we included data for three metabolic markers (cholesterol, triglycerides, and glucose) in the meta-analysis as continuous outcomes.

### Quality assessment

The quality of the selected trials was measured by Cochrane Collaboration’s tool to evaluate the risk of bias in the randomized trials (Higgins et al., 2011). The bias tools have the following respective domains: random sequence generation (selection bias), allocation concealment (selection bias), blinding of participants and personnel (performance bias), blinding of outcome assessment (detection bias), incomplete outcome data (attribution bias), selective reporting (reporting bias), and other sources of bias. Each domain was rated as a low, high, and unclear risk. If at least one of the domains showed a high or unclear risk, we classified the overall result as a high or unclear risk, respectively. The overall evaluated result was considered as low risk if all domains showed a low risk in the respective study.

### Statistical analysis

To conduct the meta-analysis, we used the review manager (RevMan) version 5.3 (Collaboration, 2016). Data in the included articles were continuous outcomes within the studies related to different metabolic markers. In the analytical method, we analyzed the random effects model by DerSimonian and Laird methods (DerSimonian and Laird, 1986). Follow-up from baseline in the experimental group was compared to that in the control group using the standard mean difference (SMD) as a primary effective measure. To identify the parametric relationship between the intervention group (wheat germ) and control group, we calculated the inverse of variance (IV) as the study weight in analysis and 95% confidence intervals (CIs) among the categories of metabolic markers. To more precisely examine the effect of cholesterol, we stratified cholesterol into subgroups: HDL-C and LDL-C.

Among the trials, some results were reported as the standard error, which was converted to standard deviation by multiplying the square root of the sample size.

Some values for triglycerides, cholesterol, and glucose were reported in mg/dL. We converted these values to units of mmol/L by dividing the values in mg/dL by a factor of 88.5, 38.6, and 18, respectively. To explore the heterogeneity in the results, *l*^2^ statistic was used for evaluation, which showed the total variation attributable to heterogeneity between studies. The results were considered significant when *p* < 0.05. Thresholds of heterogeneity of 0%, ≤ 25%, ≤ 50%, and ≤ 75% were considered as no, low, moderate, and high variations among the different outcomes.

## Results and discussion

### Studies included in the analysis

**Fig. 1:**
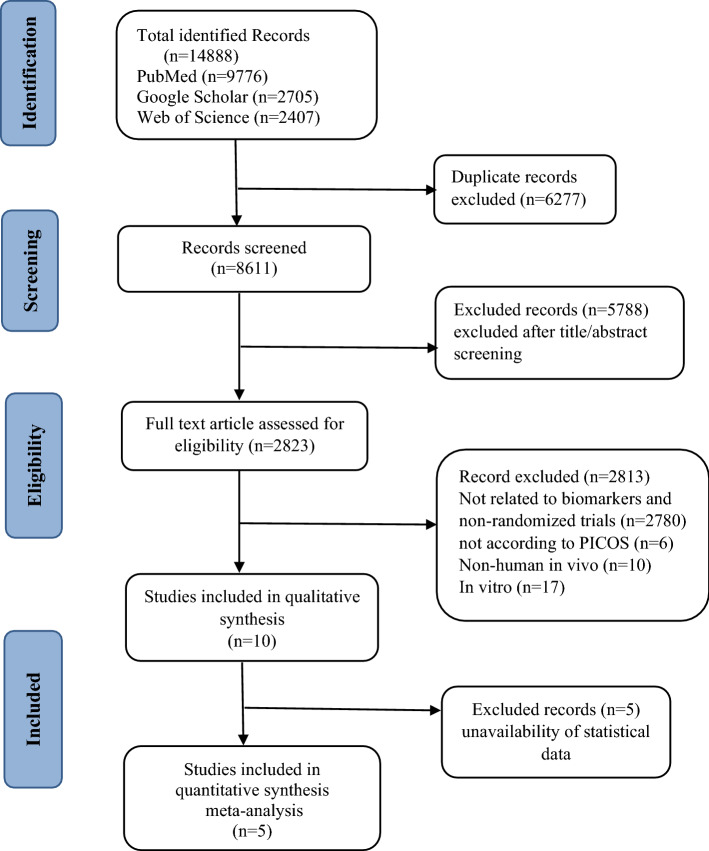
PRISMA flow chart of study identification and selection for systematic review and meta-analysis

The detailed search strategy was performed, as shown in the PRISMA flow chart (Moher et al., 2009) (Fig. 1). We initially identified 14,888 studies in the three different databases, with 9776, 2705, and 2407 articles from PubMed, Google Scholar, and Web of Science, respectively. All references from these databases were imported to an Endnote library. After deleting duplicate references using EndNote x7, 8611 studies remained. Next, 2823 full-text articles remained after eliminating abstract, proceeding, and review papers. Forty-three articles were further reviewed after eliminating 2780 studies that failed to meet the inclusion criteria. In the preparatory mapping review, we tested many studies that demonstrated the health outcomes of wheat germ consumption. Most of these studies were dropped out because of unrelated functionality and study design.

In the eligibility section, six articles were eliminated that did not evaluate whether the studies were non-randomized trials or described a diseased population (Haripriya and Premakumari, 2010; Zakaria et al., 2017). One study was also removed that did not report the functionality of wheat germ related to metabolic markers (Kobyliak et al., 2018). Finally, 10 in vivo (non-human studies) and 17 in vitro studies were eliminated. After critically reviewing the whole abstract and full-text of the articles, 10 potential studies were assessed by systematic review. However, the selection of included studies in this systematic review was challenging, because many were old studies (Cara et al., 1991; Cara et al., 1992b) with low quality RCTs (Rodionova et al., 2016). One of the studies did not report any analytical or statistical results. Still, it fulfilled the eligibility criteria of the systematic review, so it was included in the systematic review and excluded from the meta-analysis (Moreira-Rosário et al., 2016). The other four studies did not report statistical data related to metabolic markers that could be used in meta-analysis (Alessandri et al., 2006; Cara et al., 1992b; Ostlund Jr et al., 2003; Rodionova et al., 2016). Five out of ten pertinent studies describing the outcomes of metabolic markers could be used for meta-analysis (Cara et al., 1991; Cara et al., 1992b; Lin et al., 2004; Moreira-Rosário et al., 2019; Tripkovic et al., 2015). The remaining studies were excluded due to insufficient measurement of data.

### Study characteristics

The characteristics of the included studies are summarized in Table 1. Studies were conducted in different countries between 1991 and 2019. The two most recent studies were performed in a southern European country, Portugal (Moreira-Rosário et al., 2016; Moreira-Rosário et al., 2019), whereas the three oldest were performed in France (Cara et al., 1992a; Cara et al., 1991; Cara et al., 1992b). The remaining five trials were carried out in five regions: Italy (Alessandri et al., 2006), Netherlands (Lin et al., 2004), Russia (Rodionova et al., 2016), United States (Ostlund Jr et al., 2003), and United Kingdom (Tripkovic et al., 2015). The included population were mostly community-based, and some subjects were from educational institutes. Approximately half of the trials described the selected participants as healthy and four trials included slightly hypercholesterolemic populations (Alessandri et al., 2006; Cara et al., 1991; Cara et al., 1992a). Only one trial reported that the participants had risk of CVD (Tripkovic et al., 2015). The ages of participants were 18–70 years and involved approximately 320 participants in these RCTs. Although all selected studies were RCTs, six studies used a cross-over design (Cara et al., 1991; Cara et al., 1992a; Moreira-Rosário et al., 2016; Moreira-Rosário et al., 2019; Tripkovic et al., 2015) and four used a parallel design (Alessandri et al., 2006; Cara et al., 1992b; Lin et al., 2004; Rodionova et al., 2016). Included studies used wheat germ in three forms: raw, powder, and oil. Four weeks was the average intervention period in nearly all trials. The number of participants varied from 6 (Cara et al., 1992b) to 60 subjects (Rodionova et al., 2016). The wheat germ dose ranged from 3 to 80 g/day at various intervals. The sample size of each selective study was the number of subjects who participated in this analysis from baseline to follow-up with data availability.

**Table 1:** Summary of characteristics of eligible studies assessing the association between wheat germ and metabolic markers

ID	Study design	Location	Health and age	Number and gender	Biomarkers	Group: intervention and control	Dose and duration	Result
Moreira-Rosário (2019)	RCT^1^ (crossover)	Portugal	Healthy people 34 year (mean age)	55 both	Cholesterol Triglycerides Glucose	Inter^3^: WG^4^ enriched bread control: non-enriched bread	6 g/day (in two snacks) 4 weeks	× no effect
Moreira-Rosário (2016)	RCT (crossover)	Portugal	Healthy people 18–60 years	55 both	Cholesterol Triglycerides Glucose	Inter: WG enriched bread control: non-enriched bread	6 g/day (in two snacks) 4 weeks	× no effect
Rodionova et al. (2016)	RCT (parallel)	Russia	Healthy people 16–65 years	60 both	Cholesterol Triglycerides	Inter: 3.5 g/day WGO^5^ regardless of meal control: 3.5 g WGO in 50 g of meal,	3.5 oil g/day (with or without meal) 30 days	↓cholesterol ↓triglycerides
Tripkovic et al. (2015)	RCT (crossover)	United Kingdom	At risk of CVD^2^ 35–55 years	10 both	Cholesterol Triglycerides Glucose	G^6^1: WG G2: inulin G3: refined grain (control)	15 g/day (5 g/meal) 4 weeks	↓ glucose × no effect on lipid profile
Alessandri et al. (2006)	RCT (parallel)	Ital	Hypercholesterolemic 60.3 ± 6.1 years	32 both	Oxidative stress	G1: maize oil (vegetable oil) G2: WGO	1 tbsp^7^/d for 8 weeks	↓oxidative stress
Lin et al. (2004)	RCT (parallel)	Netherlands	Normal to elevated Cholesterol 18–70 years	60 both	Cholesterol Triglycerides	G1: chocolate with WG G2: chocolate without WG	20 mg/day (4 chocolates/d)	× no effect
Ostlund Jr et al. (2003)	RCT (crossover)	United States	Healthy people 39 ± 12 years	10 both	Cholesterol	G1: WG G2: WGO G3: purified WGO	80 g/day (1 muffin)	↓cholesterol
Cara et al. (1992a)	RCT (crossover)	France	Hypercholesterolemic and hyperglycemic 37–69 years	19 both	Cholesterol Triglycerides	G1: raw WG G2: partially defatted WG	20 g/day (incorporated in 3 times meal) 4 weeks	↓ cholesterol ↓triglycerides
Cara et al. (1992b)	RCT (parallel)	France	Healthy people 22–41 years	6 males	Cholesterol Triglycerides	G1: oat bran G2: rice bran G3: wheat fiber G4: WG G5: control	10 g/day total dietary fiber 2.8 g/day dietary fiber	↓ cholesterol ↓ lipid profile
Cara et al. (1991)	RCT (crossover)	France	Hypercholesterolemic and healthy subjects 35–68 years	10 both	Cholesterol Triglycerides	G1: basal diet (as control) G2: basal diet + WG	30 g/day (incorporated in meal) 4 weeks	↓ cholesterol ↓triglycerides

^1^RCT, randomized control trial; ^2^CVD, cardiovascular disease; ^3^inter, intervention; ^4^WG, wheat germ; ^5^WGO, wheat germ oil; ^6^G, group; ^7^tbsp, tablespoon

In this systematic review, many studies were older and included a very small study size with little information (Cara et al., 1991; Cara et al., 1992b). These studies did not report the mean difference, dropout compliance, and blinding (Rodionova et al., 2016).

### Risk of bias within studies

**Fig. 2:**
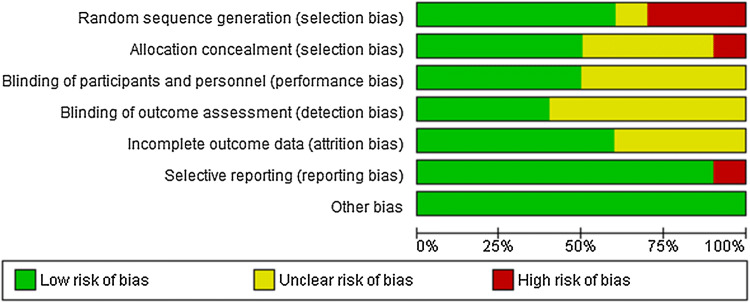
Risk of bias graph: review authors’ judgments regarding the risk of bias of each item presented as percentages across all included studies (n = 10)

The biases in the clinical trials are described in Fig. 2. Among the ten randomized controlled trials, there was a low risk of selective reporting bias and other biases. However, performance and detection bias showed more than a 50% unclear risk because of the insufficient blinding of participants and outcome assessors. There was a high risk of selection bias compared to all other domains because of the inappropriate method of selection and incomplete knowledge in many trials. The detailed risk assessment results of bias in the clinical trials are shown in Table 2.

**Table 2:** Summary of the overall risk of bias in randomized controlled trials of prospective studies in the qualitative assessment

Author (year)	Random sequence generation	Allocation concealment	Blinding of participants	Blinding of outcome assessment	Incomplete outcome data	Selective reporting	Other biases	Overall bias
Cara et al. (1991)	High risk	Unclear	Unclear	Unclear	Unclear	Low risk	Low risk	High risk
Cara et al. (1992a)	High risk	Unclear	Unclear	Unclear	Unclear	Low risk	Low risk	High risk
Cara et al. (1992b)	Low risk	Unclear	Unclear	Unclear	Unclear	Low risk	Low risk	Unclear
Ostlund Jr et al. (2003)	Unclear	Unclear	Unclear	Unclear	Low risk	Low risk	Low risk	Unclear
Lin et al. (2004)	Low risk	Low risk	Low risk	Unclear	Low risk	High risk	Low risk	High risk
Alessandri et al. (2006)	Low risk	High risk	Low risk	Unclear	Low risk	Low risk	Low risk	High risk
Tripkovic et al. (2015)	Low risk	Low risk	Unclear	Low risk	Low risk	Low risk	Low risk	Unclear
Moreira-Rosário (2016)	Low risk	Low risk	Low risk	Low risk	Unclear	Low risk	Low risk	Unclear
Rodionova et al. (2016)	High risk	Low risk	Low risk	Unclear	Low risk	Low risk	Low risk	High risk
Moreira-Rosário (2019)	Low risk	Low risk	Low risk	Low risk	Low risk	Low risk	Low risk	Low risk

In a narrative literature review, the quality of selected studies was mostly unclear and high risk. These findings should be interpreted with caution, as the results showed very low certainty of the evidence for all health outcomes.

### Effect of intervention on metabolic markers

In the primary meta-analysis, five randomized controlled trials investigated the effect of total cholesterol in 109 participants in the intervention group and 107 subjects in the control group. The SMD of the wheat germ versus control groups was − 0.03 (95% CI − 0.30 to 0.23), revealing a non-significant reduction in cholesterol (*p* = 0.69*)* and negligible heterogeneity (*I*^*2*^ = 0%).

**Fig. 3:**
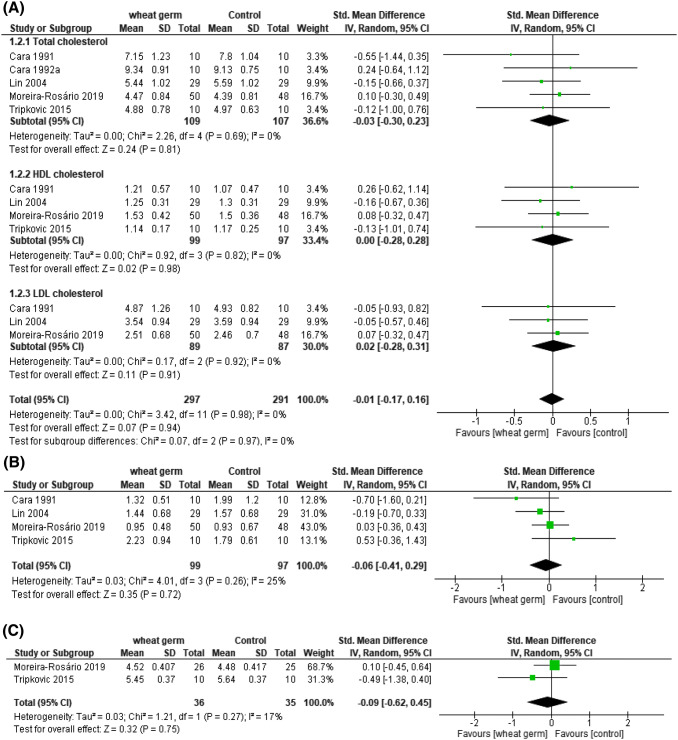
(A) Meta-analysis of wheat germ vs control group using the random-effect model and weighted by standard mean difference. Forest plot of outcome: effect of wheat germ on total cholesterol, HDL-C, and LDL-C. (B) Forest plot of wheat germ vs control: effect of wheat germ on triglycerides. (C) Forest plot of wheat germ vs control: effect of wheat germ on glucose

Cholesterol was evaluated as HDL-C and LDL-C; four trials used the HDL-C index to evaluate the SMD − 0.00 (95% CI − 0.28 to 0.28). The summary, in which the LDL-C index was used, found an SMD of − 0.02 (95% CI − 0.28 to 0.31) (Cara et al., 1991; Lin et al., 2004; Moreira-Rosário et al., 2019). Both the summaries of HDL-C and LDL-C showed the level of heterogeneity (*I*^*2*^ = 0%) with non-significant results (*p* = 0.82, 0.92, respectively). Thus, the overall results showed 0% heterogeneity with a non-significant effect (*p* = 0.98). Nearly all studies showed a non-significant effect of wheat germ intervention compared to the control group [Fig. 3(A)].

To determine the effect of wheat germ intervention on the triglycerides profile, four trials included 99 participants in the intervention group and 97 subjects in the control group. A non-significant (*p* = 0.26) reduction in triglycerides after consuming wheat germ was found, with an SMD of − 0.06 (95% CI − 0.41 to 0.29); no heterogeneity was detected (*I*^*2*^ = 0%) [Fig. 3(B)].

Two randomized controlled trials (Moreira-Rosário et al., 2019; Tripkovic et al., 2015) were added in the analysis to determine the effectiveness of wheat germ on lowering blood glucose levels. These trials included 36 participants in the intervention group and 35 in the control group. The pooled SMD was − 0.09 (95% CI − 0.62 to 0.45). The heterogeneity level measured by *I*^*2*^ was 0%, showing a non-significant result (*p* = 0.27). The meta-analysis results of these two trials were insufficient to determine whether wheat germ treatment reduces glucose levels [Fig. 3(C)]. Overall, the difference in the effects between wheat germ and control or placebo appeared to be clinically and statistically non-significant.

In summary of the evidence, our goal was to demonstrate the effect of one important part of staple food in a healthy or risk group population by systematized review of the previous literature. After performing the initial literature search on the broad topic “wheat germ” related to various health aspects, we could find only a few studies that reported the effect of wheat germ on multiple biomarkers like oxidative stress, blood cholesterol, triglycerides and glucose (that all are under the metabolic syndrome regime and more precisely, metabolic markers), and only a few studies were related to cancer, arthritis, and the immune system.

MetS is a broad term with many biomarkers, but we couldn’t find any other studies that might report on other biomarkers (obesity and blood pressure). Due to limited literature availability, our systematic review was limited to a few studies with some metabolic markers.

The strength of the current review is that only healthy and risk group individuals (slightly elevated blood lipids or glucose level) were included, rather than all diseased populations. The dietary impact varied with the usage of medications and other nutritional supplements. The metabolic rate and biomarker levels fluctuate under disease conditions; thus, evaluating only healthy participants will reveal more accurate and precise results. The data from RCTs were used rather than those obtained by other study designs. RCTs are more frequently performed in medical and clinical experiments and show low bias during testing.

Nevertheless, there were some limitations to this study. First, all eligible were studies conducted in either European countries or the United States. However, wheat is the dominant staple food in North Africa and West and Central Asia (Alexandratos and Bruinsma, 2012). Thus, the generalizability of these trials to other populations worldwide is limited. Second, there was heterogeneity in the dose, duration, and frequency of wheat germ intervention. An insufficient dose of wheat germ appeared to be among the major causes of non-significant results. A recent study proposed that a low level of wheat germ (6 g) intervention did not affect glucose and lipid metabolism (Moreira-Rosário et al., 2019). Thus, an increased dose and duration of wheat germ in the intervention group may show a preventive effect on the metabolic markers. At the last, the composition of wheat germ may be affected by factors such as the chemicals and preparation method used to treat the wheat germ. In the studies discussed here, several forms of wheat germ (raw, extracted, defatted, and oil) were added to different food commodities (chocolate pellets, bread rolls, and muffins).

In this comprehensive literature, the findings should be interpreted with caution based on the limited numbers of included studies. The review of individual studies revealed contradictory results. Some articles described the improvements in blood lipid levels (Cara et al., 1991; Cara et al., 1992a; Cara et al., 1992b; Rodionova et al., 2016) and glucose metabolism (Tripkovic et al., 2015), whereas others were unclear about the antihyperlipidemic and antihyperglycemic effect of wheat germ (Lin et al., 2004; Moreira-Rosário et al., 2016). The small intervention dose, short study duration, low study quality, some additional factors, and lack of blinding of the participants and outcomes by the assessors may have led to inconsistency among the observed results. Therefore, we could not reach the conclusion that would present any kind of quick suggestion about any significant improvement in metabolic markers with wheat germ intervention. Further relevant research is needed because these findings showed low certainty of the evidence for all health outcomes of wheat germ.

Best to our knowledge, this is the first systematic review and meta-analysis of the effects of wheat germ consumption on metabolic markers. In conclusion, there is little credible evidence for a relation between wheat germ intake and a reduced risk of metabolic markers. To evaluate the long-term effects of wheat germ consumption on the metabolic markers in humans, the well-designed randomized placebo-controlled trials with sufficient sample doses and optimal intervention duration may be worth trying.
