# Correction to: Effect of wheat germ on metabolic markers: a systematic review and meta-analysis of randomized controlled trials

**DOI:** 10.1007/s10068-020-00788-6

**Published:** 2020-07-08

**Authors:** Humna Liaqat, Eunseon Jeong, Kyeong Jin Kim, Ji Yeon Kim

**Affiliations:** 1 Department of Food Science and Technology, Seoul National University of Science and Technology, 232, Gongneung-ro, Nowon-gu, Seoul, 01811 Korea; 2 Department of Nano Bio Engineering, Seoul National University of Science and Technology, 232, Gongneung-ro, Nowon-gu, Seoul, 01811 Korea

## Correction to: Food Sci Biotechnol (2020) 29(6):739–749 10.1007/s10068-020-00769-9

The article “Effect of wheat germ on metabolic markers: a systematic review and meta-analysis of randomized controlled trials”, written by Humna Liaqat, Eunseon Jeong, Kyeong Jin Kim and Ji Yeon Kim, was originally published Online First without Open Access. After publication in volume 29, issue 6, pages 739–749 the author decided to opt for Open Choice and to make the article an Open Access publication. Therefore, the copyright of the article has been changed to © The Author(s) 2020 and the article is forthwith distributed under the terms of the Creative Commons Attribution 4.0 International License (http://creativecommons.org/licenses/by/4.0/), which permits use, duplication, adaptation, distribution and reproduction in any medium or format, as long as you give appropriate credit to the original author(s) and the source, provide a link to the Creative Commons license, and indicate if changes were made.

The original article has been corrected.

